# The TLR2 Binding Neisserial Porin PorB Enhances Antigen Presenting Cell Trafficking and Cross-presentation

**DOI:** 10.1038/s41598-017-00555-4

**Published:** 2017-04-07

**Authors:** Michael L. Reiser, Munir M. Mosaheb, Christina Lisk, Andrew Platt, Lee M. Wetzler

**Affiliations:** 1 Department of Microbiology, Boston University School of Medicine, Boston, USA; 2 Section of Infectious Diseases, Department of Medicine, Boston Medical Center, Boston, USA; 3 Division of Immunology, Department of Microbiology and Immunobiology, Harvard Medical School, Boston, USA

## Abstract

TOLL-like receptor (TLR) ligands activate both innate and adaptive immune cells, while modulating the cellular immune response. The outer membrane protein (OMP) from *Neisseria meninigitidis*, PorB, is a naturally occurring TLR2 ligand and functions as an adjuvant. Here, we demonstrate that PorB increases the level of OVA in the endo-/lysosomal cellular compartment of BMDCs, increases antigen presenting cell (APC) trafficking to draining lymph nodes, and enhances antigen cross-presentation. PorB is capable of mounting an antigen specific T cell response by efficiently stimulating antigen cross-presentation *in vivo* and *in vitro* assessed by BMDC OT-I cocultivation assays. The enhanced antigen cross-presentation and the increased APC recruitment to secondary lymphoid tissues expand the scope of known adjuvant effects of PorB on the immune system. Our findings lead to a better understanding of how TLR-ligand based adjuvants can alter and modulate immune responses.

## Introduction

Vaccines are one of the most important achievements in modern medicine within the 20^th^ century. The development of new vaccines against infectious diseases (e.g. Tuberculosis, Measles, HIV, or Ebola viruses etc.) will require a detailed mechanistic understanding of the pathogen and host interactions, including entry, pathogenesis, and early events during the innate immune response, ultimately leading to a protective adaptive immune response. An essential component of an effective vaccine is its formulation with an adjuvant. Live attenuated or inactivated whole pathogen vaccine preparations have inherent adjuvants, e.g. TOLL-like receptor (TLR) agonists, or viral nucleic acids, inducing a directed immune response. To date, adjuvants are co-administered with poorly immunogenic antigens, shaping immune responses. They act simultaneously as delivery systems, enhancers and immunomodulators. The mechanism of action for each adjuvant is very specific. Aluminum salts (Alum) for example have a well-established safety profile and have been used in millions of vaccine doses until now. Their exact mode of action, however, is still under investigation^1, 2^. Alum activates antigen-presenting cells (APCs), increases antigen uptake and recruits APCs to site of injection^3, 4^. Novel vaccination strategies include the combination and integration of TLR agonists as adjuvants or other new formulation approaches and are currently in the focus of research for more effective vaccine design^5–7^. Pathogen Associated Molecular Patterns (PAMPs), like TLR ligands, have been extensively studied as vaccine adjuvants^8–11^. They are known to function through a variety of pathways, including direct stimulation of APCs^12, 13^, increased antigen uptake and antigen processing^14^, as well as the release of inflammatory and regulatory cytokines upon APC maturation^15^. Antigen presenting cells play a major role in the onset of protective immunity. In addition to antigen uptake, the antigen needs to be proteolytically degraded, loaded onto either MHC class II or class I for CD4 or CD8 T cell stimulation, respectively, and costimulatory receptors on the APC have to be engaged to trigger activation and proliferation of T cells upon APC arrival in the draining lymph node. Dendritic cells (DCs), as professional APCs, are capable of stimulating both B cells and T cells in context of major histocompatibility complexes. Targeting antigen to DCs and the induction of cytokines aiding in the onset of a protective immune response are key features for an efficient vaccine^6, 10, 16^. Different DC subsets and other cell associated or soluble mediators determine the type of immune response, i.e. T_h1_ or T_h2_. CD8α^−^ DCs utilize MHC II for exogenously derived antigen and present it to CD4 T cells, whereas CD8α^+^ DCs present exogenous antigen in context of MHC class I to CD8 T cells, a process also called cross-presentation^17–19^.

Our laboratory utilizes the TLR2 stimulating capacity of PorB, a trimeric outer membrane protein derived from *Neisseria meningitidis* ^13, 20^ to characterize the underlying molecular adjuvant mechanisms. Many investigators, including us, have shown, that PorB acts as a potent adjuvant and induces a robust immune response, when co-administered with poorly immunogenic antigens, e.g. peptides^21^ or ovalbumin^22^. In addition, PorB has been shown to be able to induce a T cell-dependent response to normally T cell-independent antigens, like bacterial capsular polysaccharides (CPS)^23–26^. Our group has shown that PorB enhances the humoral immune response to the meningococcal CPS, which is dependent on increased expression of CD86 on APCs^27^. We also have demonstrated that PorB enhances immune responses to *Francisella tularensis* LPS, and increases protection in a mouse model of pulmonary tularemia^28^. PorB is a ligand of TLR1/TLR2 heterodimers and acts as a MyD88-dependent PAMP^22^. PorB has been used in humans as an immune adjuvant^23–26^. Yet, the complete underlying molecular mechanisms, how PorB’s adjuvant effect aids in the generation of protective immune responses, are not fully understood.

In the present study we investigated how PorB modulates antigen localization within professional APCs using *in vitro* matured bone marrow derived dendritic cells (BMDCs) stimulated with fluorescently labeled ovalbumin (OVA). PorB formulation enhanced the shuttling of incorporated antigen in the endo-/lysosomal compartment relevant for cross-presentation. *In vivo*, PorB facilitated immune cell trafficking to draining lymph nodes and induced an OVA-specific CD8 T cell response in wild type (wt) mice. The cross-presentation of soluble OVA, *in vitro*, is significantly enhanced compared to unformulated antigen. Our findings support the potential of PorB being used as a TLR based adjuvant in vaccine formulations.

## Results

### PorB increases antigen presenting cell activation and shuttling of antigen into the endo-/lysosomal compartment

**Figure 1:**
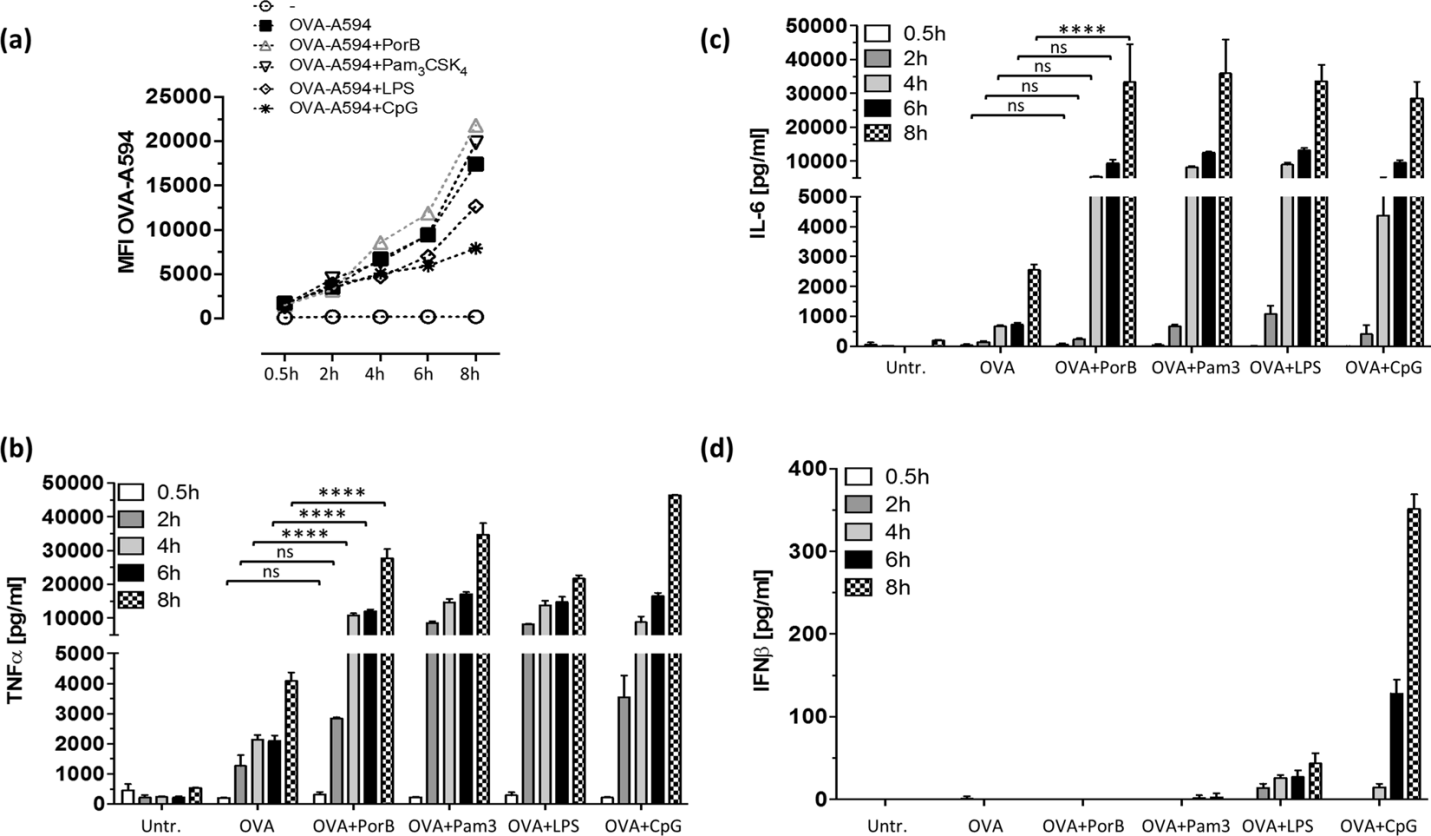
Alexa-594 fluorescently labeled OVA-A594 uptake by C57Bl/6 derived BMDCs in presence of TLR adjuvants and the respective cytokine response. **(a)** BMDCs (5 × 10^5^ cells/ml) were either left untreated (open circle, 1), stimulated with OVA-A594 alone (filled square, 2), OVA-A594 + PorB (grey triangle, 3), OVA-A594 + Pam_3_CSK_4_ (black triangle, 4), OVA-A594 + LPS (open diamond, 5) or OVA-A594 + CpG (star, 5) for 0.5 h, 2 h, 4 h, 6 h or 8 h respectively. Cells were subsequently stained with CD11c and CD11b antibodies and MFI of OVA-A594 was determined using a LSRII flow cytometer (see gating strategy Supplementary Fig. S1). **(b)** TNFα, **(c)** IL-6 and **(d)** IFNβ levels in the supernatants were determined using ELISA. Statistics for cytokine production were calculated based on a Two-way ANOVA with Sidak’s multiple comparisons test, for significance levels see Supplementary Table 1 for single comparisons. Significance levels: ns P > 0.05, *P < 0.05, **P < 0.01, ***P < 0.001, ****P < 0.0001. One out of 3 representative and independent experiments is shown.

**Figure 2:**
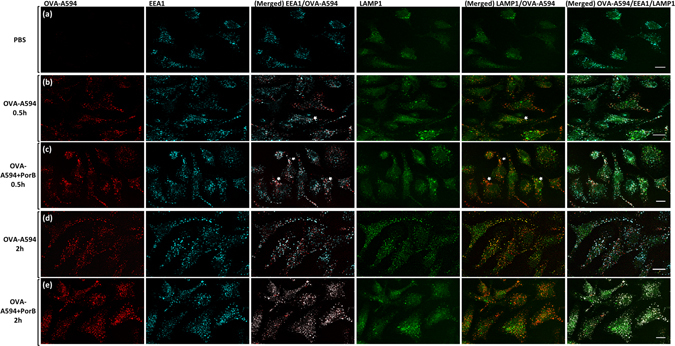
Image panel of OVA-A594 stimulated C57Bl/6 BMDCs stained for LAMP1 and EEA1. BMDCs (4 × 10^5^ cells/ml) were stimulated with PBS **(a)**, OVA-A594 **(b,d)** or OVA-A594 + PorB **(c,e)** for 0.5 h or 2 h respectively. After fixation BMDCs were stained for early endosomal antigen-1 (EEA1, cyan) and lysosomal-associated membrane protein-1 (LAMP1, green). OVA-A594 (red) localization was determined intracellularly using a Leica SP5 microscope. White arrows indicate areas of interest, where OVA colocalizes with endosomal or lysosomal vesicles. White bar represents 10 μm. One out of 3 representative experiments is shown.

**Figure 3:**
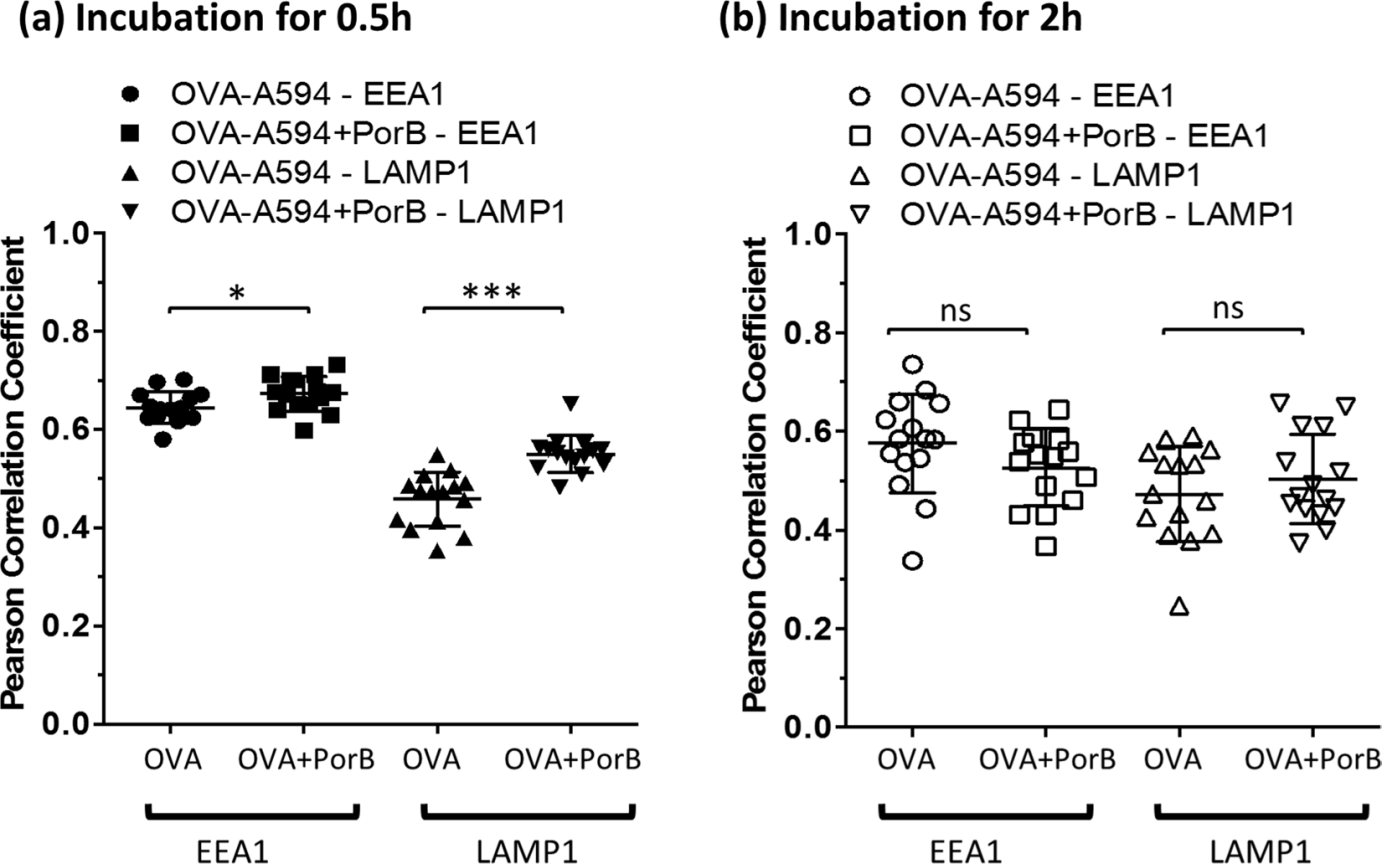
Quantification of Colocalization of fluorescently labeled OVA-A594 with intracellular EEA1^+^ and LAMP1^+^ vesicles within BMDCs. Colocalization of OVA-A594 with EEA1 and with LAMP1 was assessed using single cells. Colocalization was determined using Pearson Correlation coefficients calculated with JaCoP plugin in ImageJ, after background subtraction and unsharp mask filter. **(a)** Colocalization of BMDCs incubated for 0.5 h with OVA-A594 alone (group 1 – EEA1, group 3 – LAMP1) or PorB formulated OVA-A594 (group 2 – EEA1, group 4 – LAMP1). **(b)** Colocalization within BMDCs incubated for 2 h with OVA-A594 alone (group 1 – EEA1, group 3 – LAMP1) or PorB formulated OVA-A594 (group 2 – EEA1, group 4 – LAMP1). Statistics were calculated using the Mann-Whitney U test. Significance levels: ns P > 0.05, *P < 0.05, **P < 0.01, ***P < 0.001.

TLR ligands inherent in pathogens are known to increase antigen uptake and processing^14, 29^. We hypothesized that PorB, as a TLR2 based adjuvant, might enhance antigen uptake and simultaneously increases cytokine production to establish an inflammatory environment promoting innate and adaptive immune responses. Therefore, we investigated the influence of PorB and other TLR based adjuvants on antigen uptake by incubating wt derived BMDCs with Alexa Fluor-594 labeled ovalbumin (OVA-A594) formulated with different TLR agonists. Antigen uptake was analyzed by flow cytometry and interpreted as mean fluorescence intensity (MFI) of OVA-A594 associated with CD11c^+^ CD11b^+^ BMDCs (Supplementary Fig. S1a for gating strategy). Formulation with PorB (grey triangle) and Pam_3_CSK_4_ (black triangle, a synthesized TLR2 ligand) resulted in slightly higher MFI values, although not significant when compared to OVA-A594 alone (Fig. 1a). Formulation with CpG, a TLR9 ligand, or LPS, a TLR4 ligand, did not increase OVA-A594 uptake. We found no difference in antigen uptake in TLR2^−/−^ BMDCs (Supplementary Fig. S1b) comparing OVA-A594 + PorB with OVA-A594 only treated BMDCs. We showed previously that PorB induces inflammatory cytokines in APCs after extended exposure^13, 22^. PorB and Pam_3_CSK_4_ formulation of OVA-A594 enhanced the induction of TNFα and IL-6 significantly compared to OVA-A594 only treated BMDCs as early as 4 h for TNFα and 8 h for IL-6 following PorB stimulation (Fig. 1b,c, Supplementary Table 1 for statistics). CpG and LPS formulation also triggered high levels of TNFα and IL-6 and showed no enhanced antigen uptake. Noteworthy, PorB did not induce the type I interferon, interferon β (IFNβ), which implies that PorB does not induce signaling via IRF3 or IRF7. LPS and CpG triggered detectable amounts of IFNβ, which were significantly higher than OVA-A594 only treated BMDCs starting at 4 h for LPS and 6 h for CpG (Fig. 1d). The induced inflammatory cytokine environment triggered by PorB, led us to hypothesize that PorB formulation might rather influence antigen transfer into specific cellular compartments involved in antigen processing and presentation, than enhancing uptake for OVA as model antigen. Although exogenous antigen is usually processed and presented via the MHC class II pathway, it can also enter the MHC class I pathway, also known as cross-presentation^17–19^. This pathway requires antigen translocation and proteasomal degradation^30–32^. We therefore stimulated adherent BMDCs on glass cover slips with fluorescently labeled OVA-A594 for 30 minutes or 2 h respectively. Antigen localization was determined by antibody staining for early endosomal antigen 1 (EEA1 - Cyan) and lysosomal associated membrane protein 1 (LAMP1 - Green) using immunofluorescence microscopy (Fig. 2). PBS treated BMDCs showed no unspecific signal for OVA-A594 (Fig. 2a). PorB formulation significantly enhanced OVA-A594 colocalization within endosomes and lysosomes respectively (Figs 2c and 3a) within 30 minutes compared to OVA-A594 only treatment (Fig. 2b). Prolonged stimulation (2 h) of BMDCs with OVA-A594 with or without PorB formulation revealed increased intracellular OVA-A594 occurrence in both cellular compartments without a significant difference (Figs 2d,e and 3b). Over the course of the stimulation (0.5 h versus 2 h) the fluorescence of OVA-A594 remained clearly traceable within the endo-/lysosomal compartment and is accelerated through the prescence of PorB as early as 0.5 h. The formulation with PorB augments intracellular antigen availability in distinct cellular compartments earlier than without PorB. This potentially impacts also antigen processing, which led us to examine the effect of PorB on antigen presentation.

### PorB formulation drives cell recruitment to secondary lymphoid tissue

**Figure 4:**
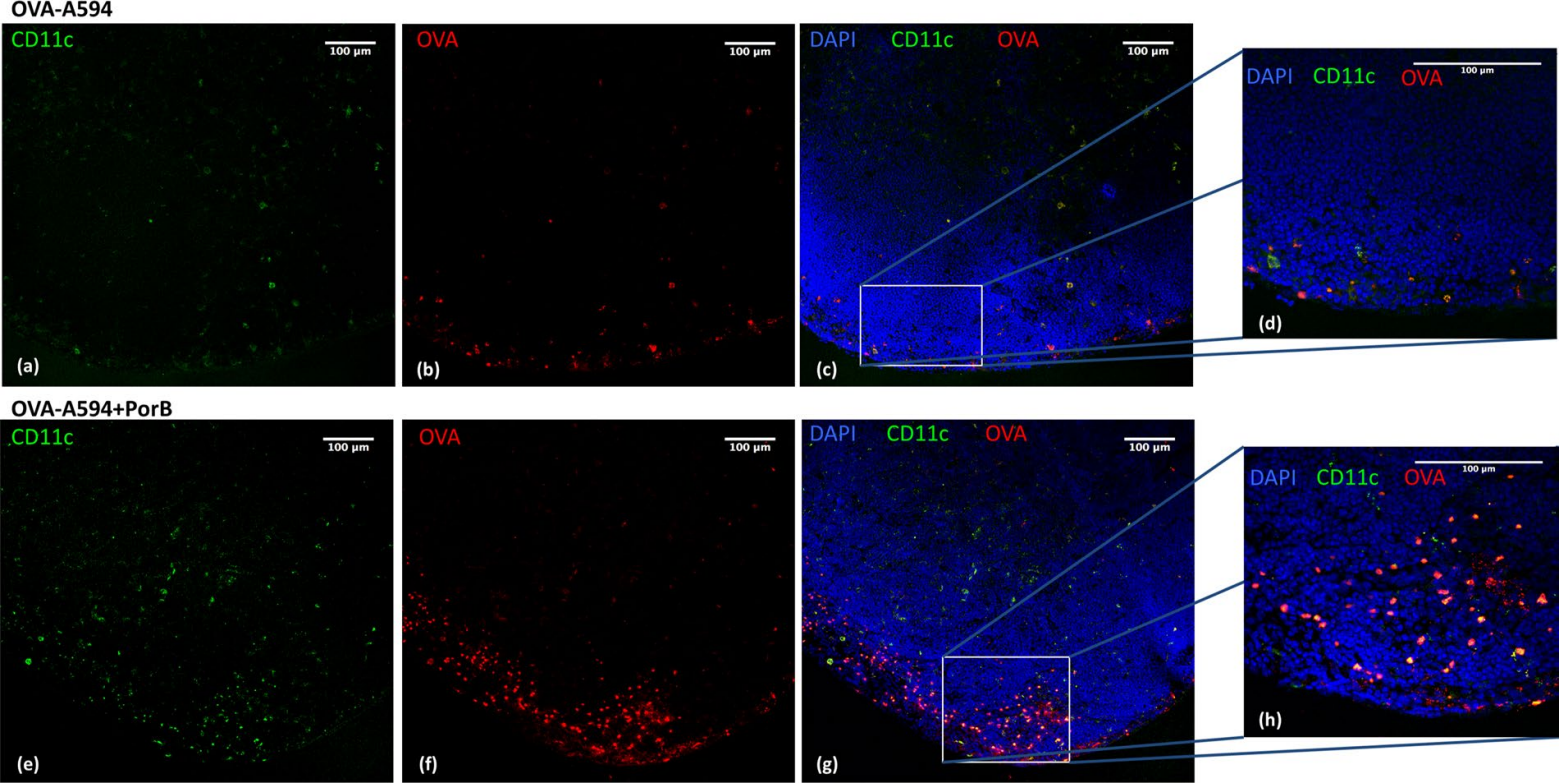
PorB formulation of OVA-A594 increases antigen bearing CD11c^+^ cells within draining popliteal lymph node. Wt mice were hock vaccinated with either OVA-A594 or OVA-A594 + PorB in a total volume of 10 μl volume. Draining popliteal lymph nodes were obtained after 16 h and sectioned (8 μm, frozen sections). Sections were stained for CD11c (green) and embedded with DAPI (blue) containing Fluoroshield mounting media. **(a–d)** Representative lymph node section of OVA-A594 injected mouse using 40x objective for magnification **(a–c)**. **(d)** Enlarged (40x objective) image of indicated area (box). **(e–h)** Representative lymph node section of OVA-A594 + PorB vaccinated C57Bl/6 mouse using 20x objective for magnification **(e–g)**. **(h)** Enlarged (40x objective) image of indicated area (box). One out of 2 representative and independent hock vaccination experiments is shown.

**Figure 5:**
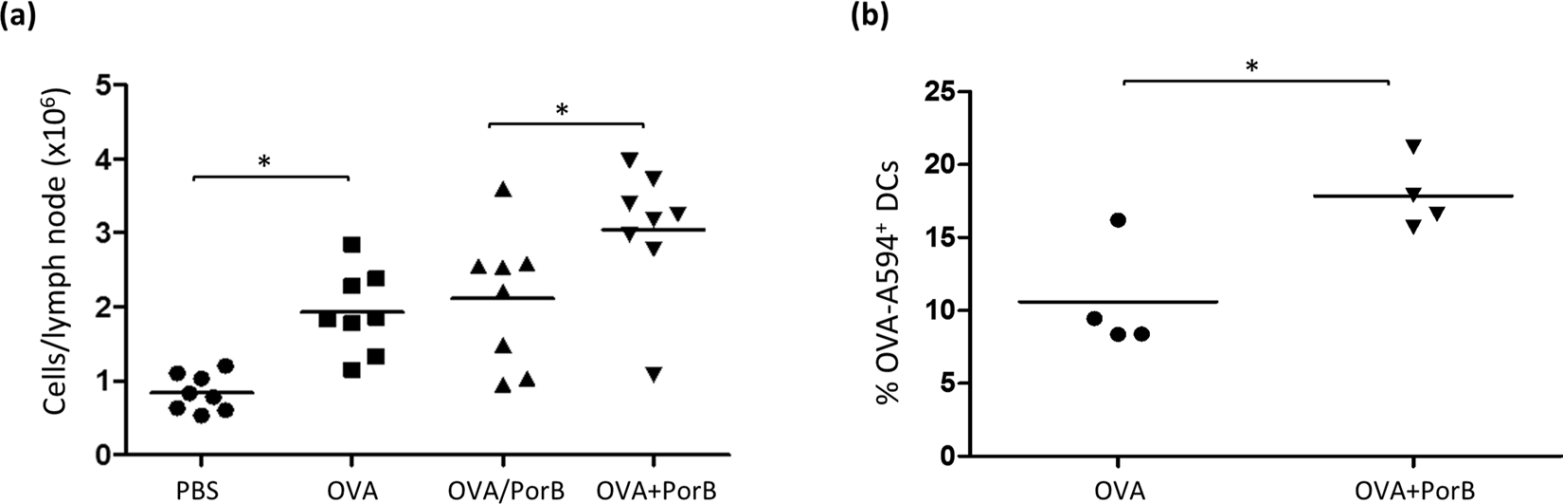
PorB increases draining lymph node cellularity and percentage of OVA-A594^+^ CD11c^+^ DCs. **(a)** Cell counts of popliteal lymph nodes of hock vaccinated wt mice. Each data point represents one lymph node. Mice were injected with PBS, OVA-A594, OVA-A594/PorB contralateral or OVA-A594 + PorB. Lymph nodes were obtained 16 h post injection and single cell suspensions were prepared. N = 8 mice per treatment. Statistics were calculated using the Mann-Whitney U test. *P < 0.05, n = 8, data represents one of two experiments. **(b)** Flow cytometry data comparing gated CD11b^−^ CD11c^+^ DCs from draining popliteal lymph nodes injected either with OVA-A594 or OVA-A594 + PorB. Statistics calculated using the Mann-Whitney U test. *P < 0.05 n = 4, data represents one of two experiments.

Antigen delivery and APC – T cell interactions within the draining lymph node are essential for the initiation of an adaptive immune response. We utilized a hock vaccination model to analyze the effect of PorB on antigen trafficking *in vivo* ^33^. Each mouse served as its own control, receiving OVA-A594 alone or formulated with PorB in one hock and PBS in the other. The antigen will drain to popliteal lymph nodes post injection, but fails to cross to the contralateral lymph node as this mechanism occurs either through vitreous pressure to the lymph or as an active event relying on antigen transport by DCs^34^. Figure 4 illustrates that PorB, as an adjuvant, significantly increased the presence of antigen loaded CD11c^+^ cells within popliteal lymph nodes (pLN) of wt mice 16 h after injection (Fig. 4e–h), whereas OVA-A594, alone, did not (Fig. 4a–d). Noteworthy, the number of CD11c^+^ cells was distinctively higher in OVA-A594 + PorB injected mice compared to mice given OVA-A594 alone. When mice were vaccinated with OVA-A594 alone or PBS in one limb, and OVA-A594 + PorB in the contralateral limb, significantly more antigen positive cells were in the draining pLN of the limb receiving OVA-A594 + PorB as compared to the contralateral pLN of the same mouse injected with PBS or OVA-A594 alone (Fig. 5a). Antigen administration with either vaccine resulted in significantly increased cell counts compared to PBS control. Furthermore, we detected markedly more antigen bearing CD11c^+^ CD11b^+^ cells using flow cytometry in single cell suspensions obtained from OVA-A594 + PorB injected mice compared to OVA-A594 alone (Fig. 5b). Control vaccination with PBS resulted in no antigen positive CD11c^+^ CD11b^+^ cells when the contralateral leg was vaccinated with OVA-A594, confirming an absence of midline crossover and the validity of our internal controls. Our findings highlight the adjuvanticity and the potency of PorB as a TLR-ligand based vaccine adjuvant in triggering immune cell trafficking into the draining lymphoid tissue.

### PorB enhances activation of OVA specific T cells *in vitro*

**Figure 6:**
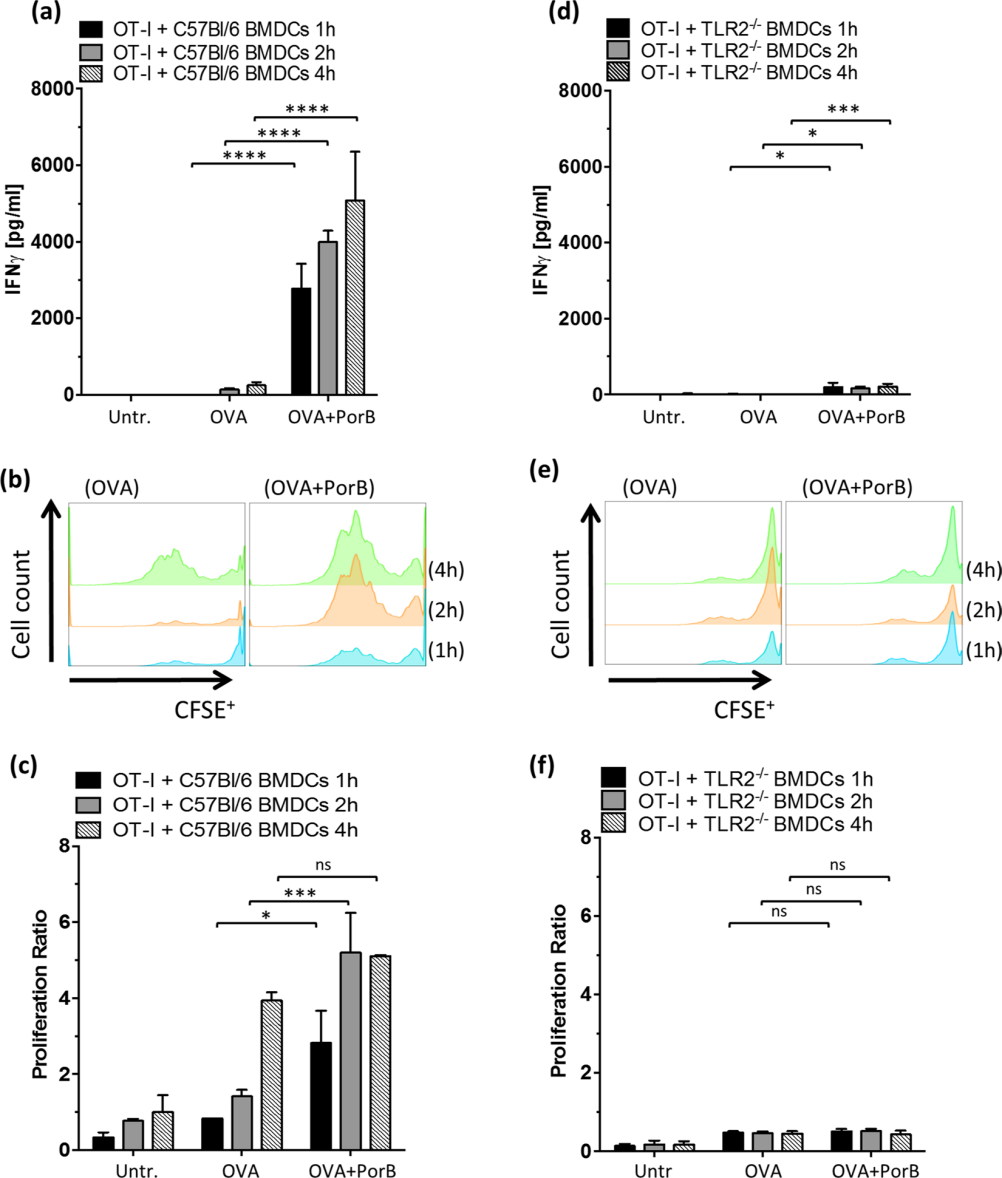
PorB enhances proliferation and cross-presentation in OT-I BMDC cocultures. *In vitro* generated BMDCs derived from wt **(a–c)** or TLR2^−/−^ mice **(d–f)** were left untreated (untr.), or were stimulated with OVA protein or OVA + PorB for 1 h, 2 h or 4 h respectively. Afterwards these BMDCs were co-incubated with CFSE stained OT-I splenocytes for 3 days in an effector to target ratio of 4:1. (**a,d**) IFNγ production was determined as a readout for efficient cross-presentation of OVA by BMDCs. OT-I T cell proliferation was assessed using flow cytometry and staining for CD8 T cells **(b,e)**. The proliferation ratio was calculated based on the quotient of CFSE^+^ proliferated CD8^+^ T cells divided by CFSE^+^ not-proliferated CD8^+^ T cells for the wt **(c)** and TLR2^−/−^ cocultures **(f)**. One out of 2 independent representative experiments shown. Statistics were calculated using a Two-way non-parametrical ANOVA with Tukey correction for multiple comparisons. ns P > 0.05, *P < 0.05, **P < 0.01, ***P < 0.001, ****P < 0.0001.

The distinct intracellular antigen localization in prescence of PorB led us to investigate, how PorB modulates and alters antigen presentation. Cross-presentation of exogenous antigen via MHC class I constitutes an important pathway to elicit a protective cytotoxic CD8 T cell response to clear intracellular pathogens or tumors^35, 36^. PAMP engagement has been shown by many investigators to facilitate and enhance cross-presentation, e.g. for intracellular nucleic acid sensors like RIG-I^37^, STING^38^ or TLR signaling via TLR3 or TLR9^39^. We hypothesized that PorB, as a TLR2 agonist might enhance cross-presentation of soluble OVA. T cells, derived from OT-I transgenic mice are a well established model to investigate MHC class I antigen recognition by antigen specific CD8 T cell in *in vitro* ^39^ and *in vivo* ^37^. OVA antigen uptake, protelytical degradation and presentation in context of MHC class I will trigger recognition of the “*SIINFEKL*” peptide - MHC class I complex on the APC by OT-I derived CD8 T cells. The cellular immune response of the CD8 T cells was determined by measuring production of interferon γ (IFNγ) upon antigen stimulation and antigen specific T cell proliferation. We cocultivated OVA or OVA + PorB treated BMDCs (1 h, 2 h or 4 h) from either wt or TLR2^−/−^ deficient mice (Fig. 6) with CFSE labeled splenocytes from OT-I mice, to test the effect of PorB formulation on cross-presentation and antigen recognition. Formulation of OVA with PorB resulted in significantly higher IFNγ production and proliferation by CFSE labeled OT-I T cells cocultivated with wt BMDCs (Fig. 6a). IFNγ levels in coculture supernatants increased with longer exposure of APCs to PorB compared to OVA only. Notably, OVA protein alone was not sufficient to trigger high levels of IFNγ in these assays, demonstrating the importance and potency of PorB formulation. In addition, we also measured the proliferation of OVA specific CD8 T cells (Supplementary Fig. S2 for gating strategy and proliferation ratio). OVA + PorB treated wt BMDCs induced significantly higher proliferation of OVA specific CD8 T cells earlier as compared to BMDCs treated with OVA alone (Fig. 6b,c). The stimulation of wt and TLR2^−/−^ BMDCs with OVA_257-264_ peptide alone and PorB formulated peptide followed by cocultivation with OT-I splenocytes revealed an significant increase in IFNγ response by OT-I splenocytes (Supplementary Fig. S4a,b) for both genotypes. PorB formulation was necessary to induce a TNFα and IL-6 cytokine response (data not shown). Our group showed previously that PorB induces inreased MHC I and CD86 expression in BMDCs^13^. This might be a very important underlying molecular mechanism of PorB enhancing antigen specific T cell activation, by increased costimulation and MHC expression (Supplementary Fig. S4). In this setup PorB exposure alone was not sufficient to induce a IFNy response in OT-I splenocytes. TLR2 signalling was crucial to trigger IFNγ production and proliferation in BMDC – OT-I cocultures (compare Fig. 6b,c and e,f and supplementary Fig. S4). We detected a slight increase in IFNγ production of OT-I derived CD8 T cells in absence of TLR2 when incubated with OVA or OVA + PorB treated BMDCs (Fig. 6e,f). We additionally tested the effect of PorB formulation on the IFNγ production of OVA specific CD4 T cells utilizing OT-II transgenic mice, recognizing a distinct CD4 T cell eptitope within OVA in context of MHC class II. PorB enhanced the antigen presentation of soluble OVA in wt BMDCs significantly with respect to IFNγ levels in OT-II cocultures compared to OVA only (Supplementary Fig. S5a), similar to our findings in the OT-I – BMDC cocultures. The IFNγ production in both OT-I and OT-II cocultivation assays, triggered by TLR2^−/−^ BMDCs, was greatly reduced and only a fraction as compared with wt derived BMDCs (Fig. 6d, supplemetal Fig. S5b). However, both genotypes, C57Bl/6 and TLR2^−/−^ derived BMDCs, exhibited increases in IL-6 and TNFα production (Supplementary Fig. S3), when PorB was present as compared to OVA alone or OVA peptide control. Cytokine levels of TLR2 deficient BMDCs were markedly less compared to C57Bl/6 derived BMDCs (Supplementary Fig. S3). The reduced proliferation in OT-I splenocytes cocultivated with TLR2^−/−^ BMDCs and the limited IFNγ response of cocultered OT-I and OT-II derived T cells confirm and strengthen the importance of TLR2 signaling in PorB’s adjuvant activity.

### PorB triggers OVA specific CD8 T cell immune response *in vivo*

**Figure 7:**
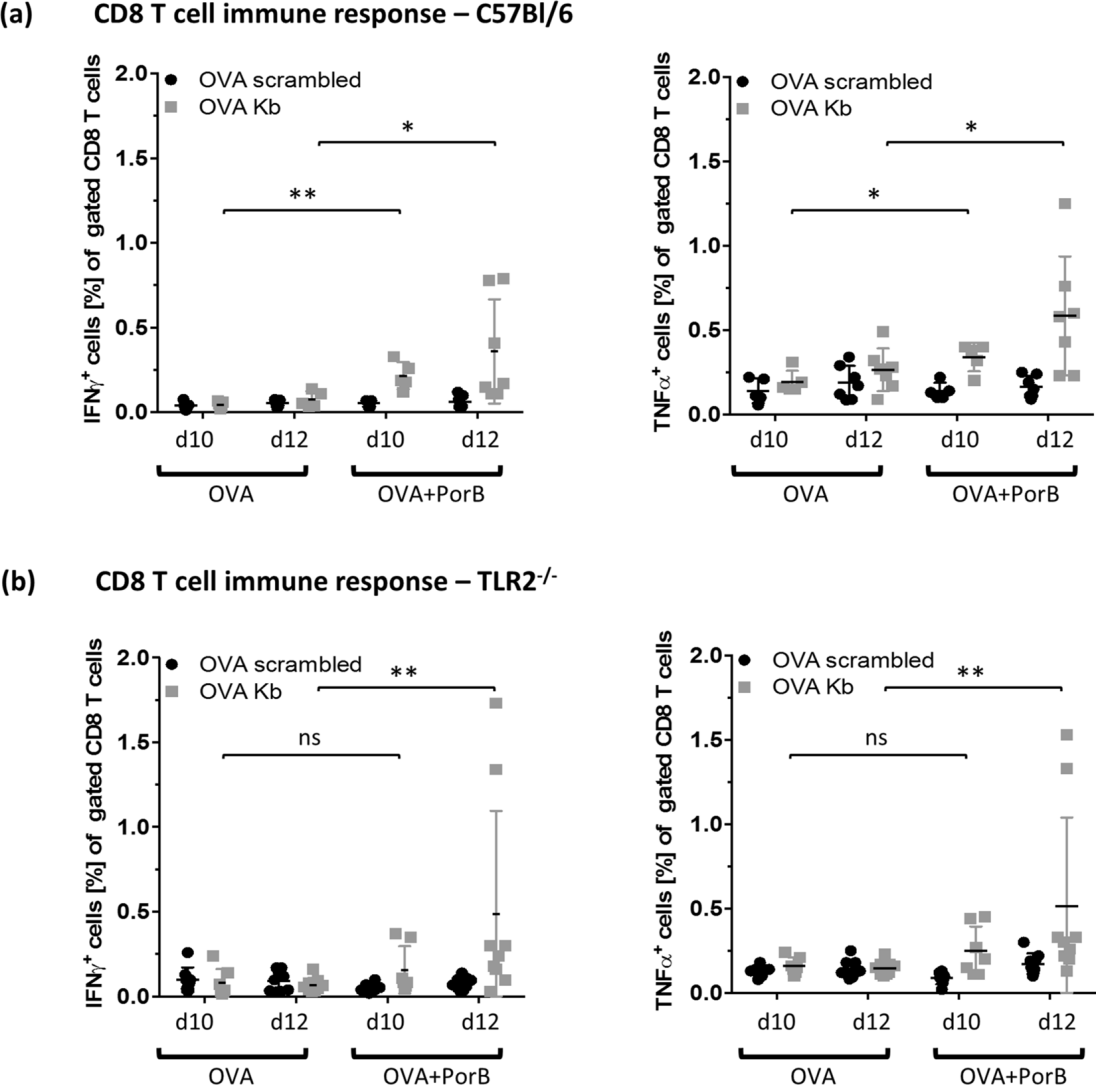
CD8 T cell response against OVA in C57Bl/6 and TLR2^−/−^ mice triggered through PorB formulation. Wt **(a)** and TLR2^−/−^ **(b)** were immunized intramuscularly (i.m.) with either OVA or OVA + PorB and received a booster vaccination 20 days later into the *tibialis anterior* muscle. The specific immune response was measured using intracellular cytokine staining of CD8 T cells 10 or 12 days post booster vaccination as indicated. OVA specific CD8 T cells frequencies were determined after 4 h *ex vivo* restimulation of splenocytes with respective peptides with intracellular IFNγ and TNFα as the readout. Two independent experiments were combined for the analysis of the specific CD8 T cell response. Statistics based on Mann-Whitney U test. ns P > 0.05, *P < 0.05, **P < 0.01.

Vaccines containing PorB are potent inducers of immune responses^21, 22^. However, the underlying T cell response has not been characterized in depth. Based on the hock vaccination model, we injected wt and TLR2^−/−^ mice, twice, intramuscularly into the *tibialis anterior* with either OVA alone or OVA + PorB as described^40^. Ten and twelve days post booster vaccination (Supplementary Fig. S6a for vaccination setup) mice were sacrificed and single cell splenocyte suspensions were re-stimulated *ex vivo* with OVA peptide or a control peptide and analyzed for the presence of intracellular IFNγ and TNFα using flow cytometry (gating strategy in supplementary Fig. S6b). We found significantly more IFNγ and TNFα producing CD8 T cells ten and twelve days post booster vaccination in wt mice immunized with OVA + PorB (Fig. 7) as compared to OVA alone. Notably, PorB was able to induce a minor amount of OVA specific CD8 T cells even in TLR2 deficient mice. OVA + PorB immunized TLR2^−/−^ mice did show significantly more IFNγ and TNFα positive CD8 T cells upon *ex vivo* stimulation with OVA_257-264_ peptide on day twelve. IFNγ and TNFα levels were comparable to those found in wt mice (Fig. 7a,c), although showing greater variability. There was no difference in the specific CD8 T cell response comparing day ten and day twelve post booster vaccination in wt and TLR2^−/−^ mice. In addition, we determined the OVA specific humoral response using IgG ELISA (Supplemetary Fig. S7). The OVA specific humoral response was significantly higher when PorB was present in the vaccine in the wt mice at day twelve post booster injection (Supplemetary Fig. S7a,b) The IgG response pattern for day ten followed the same trend (data not shown). The IgG titers in TLR2^−/−^ mice reached only about 1/10 of the IgG titers in wt mice with the OVA + PorB vaccine (compare supplemetary Fig. S7a and b) consistent with previous results published from our group^22^.

In summary, the adaptive cellular as well humoral immune response against soluble OVA formulated with PorB is significantly higher than OVA alone, even in the absence of TLR2. The increased CD8 T cell response, in both wt and TLR2^−/−^ mice, indicates that the CD8 T cell immune response triggered by PorB is partially independent of the presence of TLR2 as receptor for PorB. A similar finding has been reported for synthetic TLR2 ligands covalently fused to peptides^41^. The TLR2-independent cytokine induction might be a possible reason accounting for the ability of PorB to induce some adjuvant activity in TLR2^−/−^ mice. The residual TLR2 independent immune response, might be attributable to PorB’s particulated and aggregated form (Supplementary Fig. S8), which could enhance antigen availability in the draining lymphatics similar to virus like particles (VLPs) and nanoparticles^42^.

## Discussion

The inherent adjuvant effect in most attenuated or inactivated vaccine preparations is mainly attributable to the involvement of Pattern Recognition Receptors (PRRs) recognizing PAMPs^43, 44^. Poorly immunogenic subunit vaccines rely on the formulation with adjuvants to induce humoral as well as cellular protective immune responses. PRR agonists, as conserved PAMPs, are becoming increasingly attractive adjuvants. They are used in experimental vaccine formulations and are currently in the focus of intense research^2^. TLR agonists, as one example for PRR ligands, are capable of modulating and shaping the resulting immune response, based on their receptor usage and admixed non-TLR adjuvants. The triggered immune response can include humoral responses, T-helper 1 and 2 (T_h1_, T_h2_) responses, and cytotoxic CD8 T cell responses or combinations thereof. Lipid A analogues, e.g. MPLA, bind to TLR4 and trigger a specific antibody and T_H_1 directed immune response^45^. CpG ODNs in contrast, stimulate via TLR9 and vaccine antigen formulation with CpG results in humoral, T_h1_ directed, and CD8 T cell response^46, 47^. Outer Membrane Protein Complexes (OMPCs) isolated from *Neisseria meningitidis* were used as carrier for *Haemophilus influenza B* (Hib) capsular polysaccharide as part of the Hib-OMPC vaccine^23^ and induced immune responses with T_h2_ characteristics, relying on T cell help. PorB, a known TLR2 ligand with adjuvant activity, is one of the major constituents of this vaccine. In the present study we examined the effect of purified PorB on intracellular antigen shuttling, antigen-presenting cell trafficking to draining lymph nodes and the antigen specific T cell immune response of the well-characterized model antigen ovalbumin, as a measure of PorB’s adjuvant activity. Our findings extend the knowledge on how PorB acts as potent adjuvant and immunomodulator.

Our group previously demonstrated that PorB induces inflammatory cytokines and the presence of TLR2 was crucial for PorB to act as an adjuvant^27, 28^. TLR2 agonists have been described to favor antigen uptake and alter the immune response outcome^48, 49^. TLR2 also, recognizes ligands in combination with TLR1, TLR6 or TLR10 (in humans) as heterodimers leading to a wide range of ligands such as lipoproteins, lipopeptides or peptidoglycans. The downstream signaling involves MyD88 as an adaptor protein. Pam_3_CSK_4_, a synthetic TLR2 agonist, enhances antigen uptake and cross-presentation of apoptotic cells^50^. Subunit vaccines composed of a synthetic TLR2/TLR6 ligand, namely Pam_2_Cys, covalently linked to CD4 T cell epitopes and target epitopes, triggered humoral as well as cellular immune responses^51^ strengthening the role for TLR2 agonists as effective adjuvants. In this study we utilized purified PorB, which stimulates cells via TLR2/1 and was isolated from a mutant *Neisseria meningitidis* strain lacking Rmp and PorA to minimize protein contamination. Furthermore a distinct method of preparation was used to eliminate possible LPS contamination^52^. The vaccine itself is generated by simply mixing PorB, formulated into protein nanoparticles, termed proteosomes^21, 53^ with the target antigen, these preparations do not require covalent linkage of antigen and adjuvant. This makes PorB an ideal adjuvant candidate targeting TLR2. However, PorB did not significantly increase the antigen uptake of fluorescently labeled OVA formulated with PorB, but accelerated the presence of antigen in the endo-/lysosomal compartment. It rather modulates and accelerates the antigen shuttling. In light of that, we show that PorB triggers an inflammatory cytokine response in BMDCs as early as 2 h of stimulation in the coincubation assays and enhances the antigen localization within the endo-/lysosomal compartment (Supplementary Fig. S3 and Fig. 2). The endo-/lysosomal localization of antigen is particularly important for the resulting immune response, which needs prior antigen processing by APCs and costimulation of either CD4 T cells or CD8 T cells via MHC. PorB, as a TLR2 agonist enhances the antigen targeting to endosomes (Figs 2 and 3). Together with the increase in costimulatory molecules and surface MHC^13^, this leads possibly to improved antigen presentation and increased here CD8 and CD4 T cell activation (Fig. 6 and supplementary Figs S4 and S5). Similarily, a TLR2 binding antibody was shown to augment the shuttling of antigen into the MHC II processing cellular compartment and enhance CD4 T cell activation^54^.

The exact molecular mechanisms by which PorB facilitates the antigen uptake and transport into endosomes are still under investigation and not fully clarified. Endocytosis mediated by Pam_3_CSK_4_ as part of a conjugate vaccine has been shown to be clathrin- and/or caveolin dependent^41^. This could also be attributable to PorB mediated endocytosis. PorB might utilize two distinct mechanisms for antigen uptake and intracellular processing to induce immune responses similar to what was shown for Pam_3_CSK_4_^41^_._ We cannot exclude that PorB interacts with other intracellular receptors and/or enhances the transport of OVA into phagolysosomes for efficient antigen processing/-presentation on MHC II^14^ and/or MHC I^55^.

Once stimulated, APCs will conceivably enhance their cellular trafficking to draining lymph nodes and initiate crosstalk with cells from the adaptive immunity^41^. Interaction between APCs and T cells occurs in the peripheral lymphoid tissue and requires prior antigen transport to the lymph node^56^. To investigate antigen trafficking, we utilized a hock vaccination system^33^, which yielded increased cellularity and triggered trafficking of antigen bearing CD11b^−^ CD11c^+^ DCs to the popliteal lymph node within 16 h (Figs 4 and 5) after OVA-A594 + PorB injection. We utilized APC – T cell costimulation assays to measure CD8 and CD4 T cell activation, in order to characterize their interaction. Previously, our lab showed PorB-induced APC activation via upregulation of cellular activation markers and costimulatory molecules, e.g. MHC I, MHC II, CD40, CD54, CD69 or CD86 in different APC types^13, 22, 52^. We have also shown that PorB formulated vaccines with OVA trigger a balanced T_h1_/T_h2_ immune response^13, 22^ inducing proliferation of antigen specific B cells^57^. In this report we provided evidence that PorB enhances *in vitro* antigen presentation to CD4 and CD8 T cells (Supplementary Fig. S5, Fig. 6). An OVA specific CD8 T cell activation/response requires presentation of OVA in context of MHC I, which we demonstrate in Figs 6 and 7. *In vitro*, PorB induced minimal cross-presentation of OVA in the absence of TLR2 (Fig. 6), but a significant inflammatory cytokine response (Supplementary Fig. S3). *In vivo*, we show that PorB is capable of inducing antigen specific CD8 T cells, measured by INFγ and proliferation, and increased IgGs, which were reduced but not totally absent when TLR2 was not present (Supplementary Fig. S7 and Fig. 7). A potential underlying reason for the discrepancy of slightly diminished CTL response and reduced humoral response in wt versus TLR2^−/−^ mice might be due to the particulate nature of PorB administered as an adjuvant^52, 53^. Subjected to non-denaturing polyacrylamide gel electrophoresis (PAGE), PorB proteosomes break down, due to the electric charge and appear as multimers, reflecting the molecular size of about 120–250 kDa with the trimeric PorB form most prominent at 120 kDa (Supplementary Fig. S8a). On a denaturing SDS-PAGE PorB trimers break down and the monomeric form of *Neisseria meningitidis* PorB runs at 39 kDa (Supplementary Fig. S8b). The nanoparticulate structure of PorB proteosomes could be one reason for the TLR2 independent induction of cytokines of CD8 T cells *in vivo* and *in vitro* and needs further investigation. The PorB proteosomes are larger protein aggregates composed of trimeric PorB and vary in size from 10 nm to 20 nm. The particulate nature of a vaccine greatly enhances its trafficking efficacy and the resulting immune response^42, 58^, this might be also applicaple to PorB proteosomes which are basically nanoparticles and TLR agonists at the same time.

In summary, we have demonstrated that PorB acts through two innate immunity pathways not previously reported in the adjuvant activity of PorB, namely APC trafficking and antigen cross-presentation. PorB as particulate and soluble adjuvant can modulate both pathways simultaneously and enhances the immune response outcome. This represents an essential mechanism of action for PorB and will be important in the development of next-generation vaccine adjuvants, especially for potential combination with new adjuvants.

## Materials and Methods

### Mice

Six-week-old female C57Bl/6 J (referred to as wt and C57Bl/6, stock #000664) mice, B6.Cg-Tg(TcraTcrb)425Cbn/J (referred to as OT-II, stock #004194) and C57BL/6-Tg(TcraTcrb)1100Mjb/J (referred to as OT-I, stock #003831) were obtained from Jackson Laboratories (Bar Harbor, ME). TLR2^−/−^ mice^59^ (on the C57Bl/6 genetic background) were a gift from Dr. S. Akira (Research Institute for Microbial Diseases, Osaka University, Osaka, Japan). All mice were maintained within the Laboratory Animal Science Center (LASC) at Boston University School of Medicine and used at 8–16 weeks of age. The Boston University Institutional Animal Care and Use Committee (IACUC) approved all research conducted using animal models. Experiments were conducted in accordance to IACUC guidelines.

### Generation of Bone Marrow Derived Dendritic cells (BMDCs)

BMDCs were generated from both femurs and tibiae of C57Bl/6 and TLR2^−/−^ mice^60^. Briefly, single cell suspensions were generated flushing bones using a 25 G needle and filtered through a 70 µm nylon mesh (ThermoFisher Scientific, USA). Erythrocytes were lysed with ACK lysis buffer (150 mM NaH_4_Cl, 50 mM KHCO_3_) and washed in PBS. Afterwards cells were counted and plated at a density of 3 × 10^5^ cells per ml in non-coated petri dishes, supplemented with 20 ng/ml murine rGMCSF (Sigma-Aldrich, #SRP3201) containing RPMI-1640 with 10% FCS (Gibco), 100 U/ml Penicillin-Streptomycin (Sigma-Aldrich) (referred to as R-10). Four days post generation 5 ml 20 ng/ml murine rGMCSF containing R-10 media was added to cell culture. Non-adherent BMDCs were harvested and pelleted on day 6 for antigen uptake assays and treated as indicated.

### Vaccination of mice

Immune cell trafficking to draining lymph nodes was investigated using a hock vaccination model as described^33^. Briefly, wt mice were restrained and 10 μl of formulated vaccine (PBS, 5 μg OVA-A594 alone and 5 μg OVA-A594 + 10 μg PorB) was injected into the lateral aspect of the ankle, avoiding all major blood vessels. One group (n = 8) received PBS (control) in one ankle and OVA-A594 in the second. The other group (n = 8) of the mice was vaccinated with OVA-A594 in one ankle and OVA-A594/PorB in the second. For determination of OVA specific T cell responses C57Bl/6 and TLR2^−/−^ (n = 3–4) were immunized into the *tibialis anterior* muscle as described previously^40^. Mice received two vaccinations 20 days apart with either 10 μg OVA alone or OVA formulated with 10 μg PorB. Mice were sacrificed 10 respectively 12 days post booster vaccination and splenocytes were *ex vivo* stimulated with peptides as indicated. Mice were bled prior to prime, before booster and terminally to determine OVA specific immunoglobulin levels.

### Determination of OVA specific T cell response

Single splenocyte cell suspensions were stimulated with peptides in UltraCulture (Lonza, #12-725 F) as described previously^61^. Briefly, splenocytes (5 × 10^5^/100 μl) were pulsed for 4 h with 2.5 μg/ml peptides purchased from Anaspec (USA) in the presence of 5 μg/ml brefeldin A (Sigma-Aldrich, #B7651-5mg) in serum free media. (Peptide list: control peptide OVA_scrambled_ (FILKSINE) or OVA_257-264_ (SIINFEKL) CD8 T cell peptide). Afterwards cells were washed in FACS buffer (PBS, 0.5% BSA, 0.05% sodium azide) and incubated with CD16/CD32 F_c_ receptor block for 15 minutes at 4 °C (eBioscience, USA). Samples were stained with CD8 – APC-Cy7 antibodies for 20 minutes. Surface stained cells were fixed with 2% paraformaldehyde and afterwards permeabilized with permeabilization buffer (PBS, 0.5% BSA, 0.5% saponin, 0.05% sodium azide) for 15 minutes at RT. Intracellular cytokine staining was performed with anti-IFNγ – FITC and anti-TNFα – APC fluorescent conjugate antibodies for 30 minutes at RT in permeabilization buffer and afterwards washed twice with FACS buffer. Cells were resuspended in PBS supplemented with 0.5% BSA. Data acquisition was performed on a BD LSRII and afterwards analyzed using FlowJo (version 10.0.8).

### Cytokine ELISA

Cytokine levels in supernatants of stimulated or cocultivated cells were determined as follows: TNFα and IL-6 levels in supernatants of stimulated BMDCs were determined using the Mouse TNF alpha ELISA Ready-SET-Go kit (eBioscience, #88-7324-88) and IL-6 using the Mouse IL-6 DuoSet ELISA (R&D Systems, #DY406), according to manufacturer’s recommendation. Interferon β (IFNβ) levels in supernatants were analyzed using a customized sandwich ELISA as described^62^. Briefly, 2HB plates were coated with monoclonal IFNβ antibody (0.1 μg/ml) (Santa Cruz, #sc-57201) in carbonate buffer. Samples and known IFNβ standard concentrations (PBL assay science, #12405-1) were incubated over night after blocking wells for 2 h with PBS/BSA 1%. IFNβ was detected using an anti-mouse IFNβ, polyclonal rabbit IgG (1:2000), (PBL assay science, #32400-1) for 2 h and donkey anti-rabbit IgG antibody, HRP conjugated (1:2000) (EMD Millipore, #AP182P) for 1 h. BD HRP substrate (BD, #555214) was used to develop ELISA at 450 nm. Interferon gamma (IFNγ) was measured using BD OptEIA Elisa (BD, #555138) sandwich ELISA. OD values for TNFα, IL-6, IFNβ and IFNγ were determined at 450 nm and calculated based on standard curves generated by known amounts of recombinant TNFα, IL-6, IFNβ and IFNγ using GraphPad Prism (version 6.0).

### CFSE proliferation assay

OT-I splenocytes were stained with 5 μM CFSE (LifeTechnologies, USA) and cocultivated with stimulated BMDCs for 3 days at an effector target ratio of 4:1 in 96 U-well plates. APCs were stimulated prior to coculture with 10 μg OVA alone or formulated with 10 μg PorB. BMDCs were treated with 50 μg/ml Mitomycin C (Fisher Scientific, USA) for 30 minutes and washed twice with PBS prior to coculture setup. Activation of BMDCs was verified using TNFα and IL-6 ELISA as described above. Supernatant of OT-I and OT-II BMDC cocultures were analyzed using IFNγ ELISA as described above. Proliferation of OT-I CD8 T cells was assessed using flow cytometry. Briefly, cells were stained with LD stain (Zombie Aqua, Biolegend, USA) for 20 minutes and afterwards treated with CD16/CD32 F_c_ block (as describe above). Samples were stained with CD8-APC-Cy7 and CD4 – PE and analyzed on a BD LSRII.

### Immunofluorescence imaging of pulsed BMDCs and lymph node sections

*In vitro* matured BMDCs (4 × 10^5^ cells/ml) were seeded on day 5 on fibronectin coated glass cover slips (Neuvitro, Germany) and pulsed in 24 well plates. Cells were rested over night in rGMCSF containing R-10 media and allowed to adhere prior to stimulation on day 6 with OVA-A594 [5 μg/ml] admixed with or without PorB [10 μg/ml] for 30 minutes or 2 h respectively. Afterwards cells were fixed in 2% PFA for 10 minutes at RT and permeabilized using 0.1% Triton X-100 (MP Biomedicals, USA) for 10 minutes. Cells were washed in PBS and block was performed for 30 minutes using PBS-BSA (5%, Sigma-Aldrich, USA). BMDCs were stained with primary antibody against early endosomal antigen 1 (EEA1, anti-mouse raised in goat, Santa Cruz, USA) and lysosomal-associated membrane protein1 (LAMP1, anti-mouse raised in rat, Biolegend, USA) for 45 minutes in PBS/BSA (1%). After 4 washes with PBS/BSA (1%) cells were stained for 1 h with secondary antibodies at a dilution of 1:400 (anti-goat-Alexa-647, anti-rat-Alexa-488; Life Technologies, USA). Coverslips were mounted onto slides after extensive washing with PBS using DAPI SlowFade Gold Mountant media (Invitrogen, USA). Pictures were acquired on a Leica TCS SP5 confocal microscope at the Cellular Imaging Core at the Boston University, equipped with a 405 nm diode, an argon laser and laser lines for 488 nm, 594 nm and 633 nm allowing simultenous acquisition of up to 4 channels with the Leica Application Suite Advanced Fluorescence (LAS AF) software. Image acquisition of BMDCs was performed, using the 63x oil immersion objective. Images were captured with 4 lines average at 200 Hz with a resolution of 1024 × 1024. Colocalization analysis was performed using ImageJ (NIH) as described^63^. Briefly, three representative images were selected and five cells per image were selected individually using the lasso tool. The background was subtracted for each cell separately using a rolling ball radius of 20.0 pixels. Afterwards an unsharp mask filter was applied (radius: 1.8 pixels, maskweight: 0.6). Colocalization was determined using the JaCoP plugin in ImageJ calculating the Pearson Colocalization Coefficient^64^.

Popliteal lymph node sections were obtained 16 h after hock immunization and embedded in Optimal Cutting Temperature (OCT) medium (Richard Allan Scientific) in molds (ThermoFisher). Samples were frozen in an ethanol and dry ice mixture and stored at −80 °C. Sectioning was performed on a Microm HM 550 (Microm International GmbH, Germany). 8 μm sections were obtained and placed on Colorfrost Plus slides (ThermoFisher, USA) and stored at −80 °C until staining. Sections were air dried for 15 minutes at room temperature, then fixed in acetone at −20 °C for 10 minutes and air dried for 10 minutes. Sections were re-hydrated in TBS buffer with 0.05% Tween-20 (TBS-T) then blocked for 20 minutes at room temperature with TBS-T with 5% BSA. Sections were rinsed with TBS-T and then stained with antibodies for 1 h followed by two rinses with TBS-T and incubated in a TBS-T bath for 5 minutes on an orbital shaker. The following antibodies and reagents were used: APC hamster anti-mouse CD11c (BD) and OVA-A594 (Invitrogen, USA). Stained sections were mounted in Fluoroshield mounting medium with DAPI (Abcam) and dried overnight. A Leica SP5 confocal microscope (Leica AG) was used to examine the sections using the Leica LAS AF software as described above using the 40x and 63x oil immersion objectives. The images were arranged using ImageJ (NIH).

### Statistics

Statistics were calculated in GraphPad Prism (version 6.0). Differences in cytokine production by stimulated C57Bl/6 BMDCs have been calculated using Two-way non-parametric ANOVA with the Sidak test for multiple comparisons. Significance levels for cocultivation assays and humoral immune response were determined using Two-way non-parametric ANOVA with Tukey correction for multiple comparisons. Colocalization analysis was assessed using Pearson Correlation coefficients calculated as described above. Differences in *in vitro* antigen colocalization, *in vivo* cell counts, antigen positive DCs in draining lymph nodes and specific CD8 T cell responses in vaccinated mice were calculated using the non-parametric Mann-Whitney U test. Significance Levels were: n.d. below threshold, ns P > 0.05, *P < 0.05, **P < 0.01, ***P < 0.001, ****P < 0.0001.

## Electronic supplementary material

Supplementary Information
