# Supplementary material for: The TLR2 Binding Neisserial Porin PorB Enhances Antigen Presenting Cell Trafficking and Cross-presentation
Source: Sci Rep. 2017 Apr 7;7:736. doi: 10.1038/s41598-017-00555-4 (PMC5428659; doi:10.1038/s41598-017-00555-4)
Supplement: Supplementary file 1 — Supplementary Information [file 41598_2017_555_MOESM1_ESM.pdf]

Supplementary Figures and Supplementary Protocols

The TLR2 Binding Neisserial Porin PorB Enhances Antigen Presenting Cell Trafficking and Cross-presentation

Michael L. Reiser[2], Munir M. Mosaheb[1,3], Christina Lisk[2], Andrew Platt[1], Lee M. Wetzler[1, 2*]

[1]Department of Microbiology, Boston University School of Medicine, Boston, USA

[2]Section of Infectious Diseases, Department of Medicine, Boston Medical Center, Boston, USA

[3]Present adress: Division of Immunology, Department of Microbiology and Immunobiology, Harvard Medical School, Boston, USA

*lwetzler@bu.edu

(a) Gating strategy

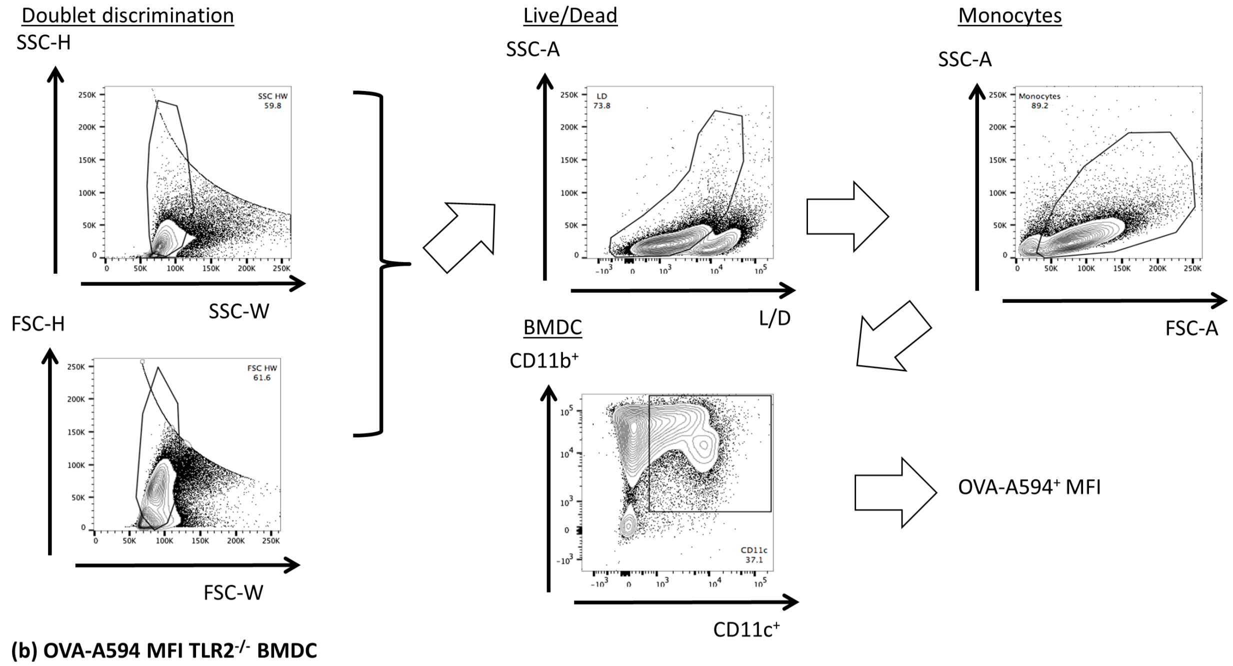

(b) OVA-A594 MFI TLR2$^{-/-}$ BMDC

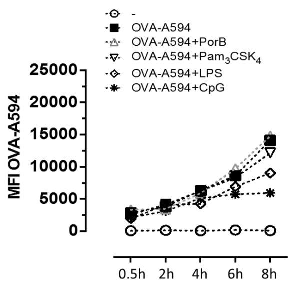

Supplemental Figure S1. Flow cytometry gating strategy for OVA-A594 MFI assessment.

(a) C57Bl/6 and TLR2$^{-/-}$ derived BMDCs (5x10^5 cells/ml) were stained with CD11c-APC and CD11b-APC/Cy7 antibodies. OVA-A594 MFI of CD11b$^+$ CD11c$^+$ DCs was determined and depicted. **(b)** TLR2$^{-/-}$ BMDCs were either left untreated (open circle, 1), stimulated with OVA-A594 alone (filled square, 2), OVA-A594+PorB (grey triangle, 3), OVA-A594+Pam$_3$CSK$_4$ (black triangle, 4), OVA-A594+LPS (open diamond, 5) or OVA-A594+CpG (star, 5) for 0.5h, 2h, 4h, 6h or 8h respectively. Cells were subsequently stained with CD11c and CD11b antibodies and MFI of OVA-A594 was determined using a LSRII flow cytometer.

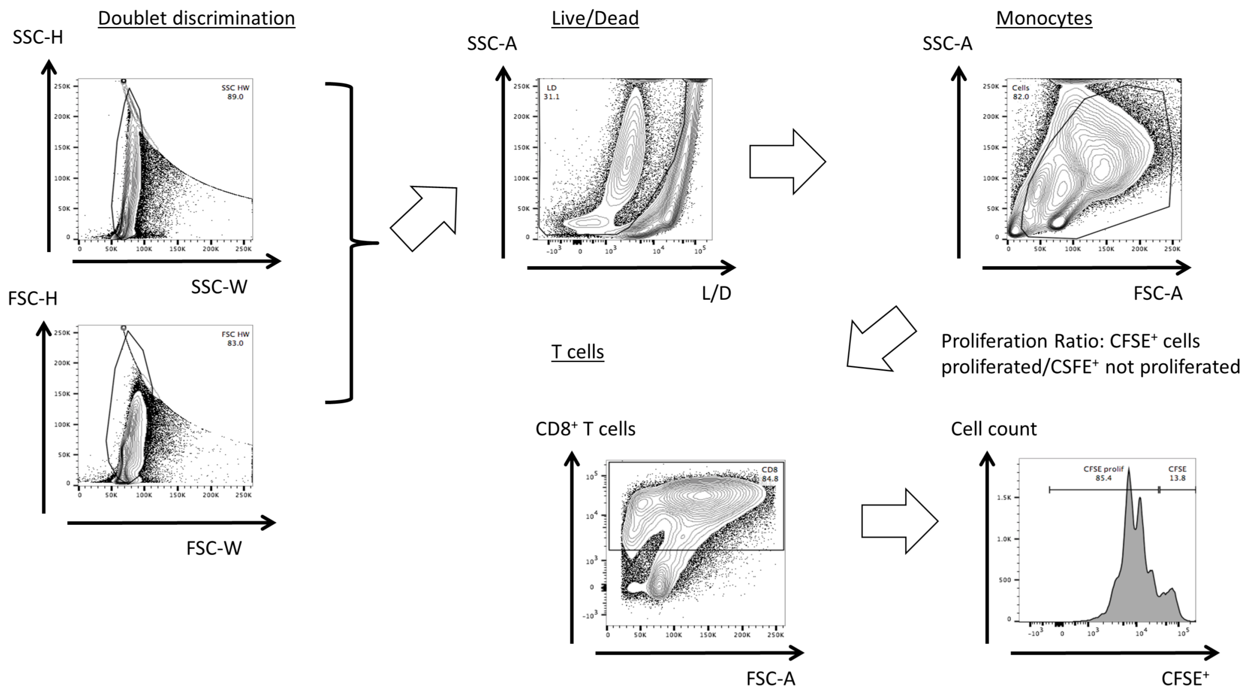

Supplemental Figure S2. Gating strategy for CFSE stained OT-I splenocytes and BMDC costimulation assay.

Gating strategy of CFSE labeled OT-I cells and assessment of proliferation ratio. OT-I – BMDC cocultures (E:T ratio 4:1) were stained after 3 days with CD8 – antibody. Proliferation index was calculated based on the quotient of “CSFE^{+} proliferated” divided by “CSFE^{+} non proliferated” cells. CFSE profile of OVA$_{257\text{-}264}$ peptide stimulated OT I derived splenocytes served as positive control and is shown here.

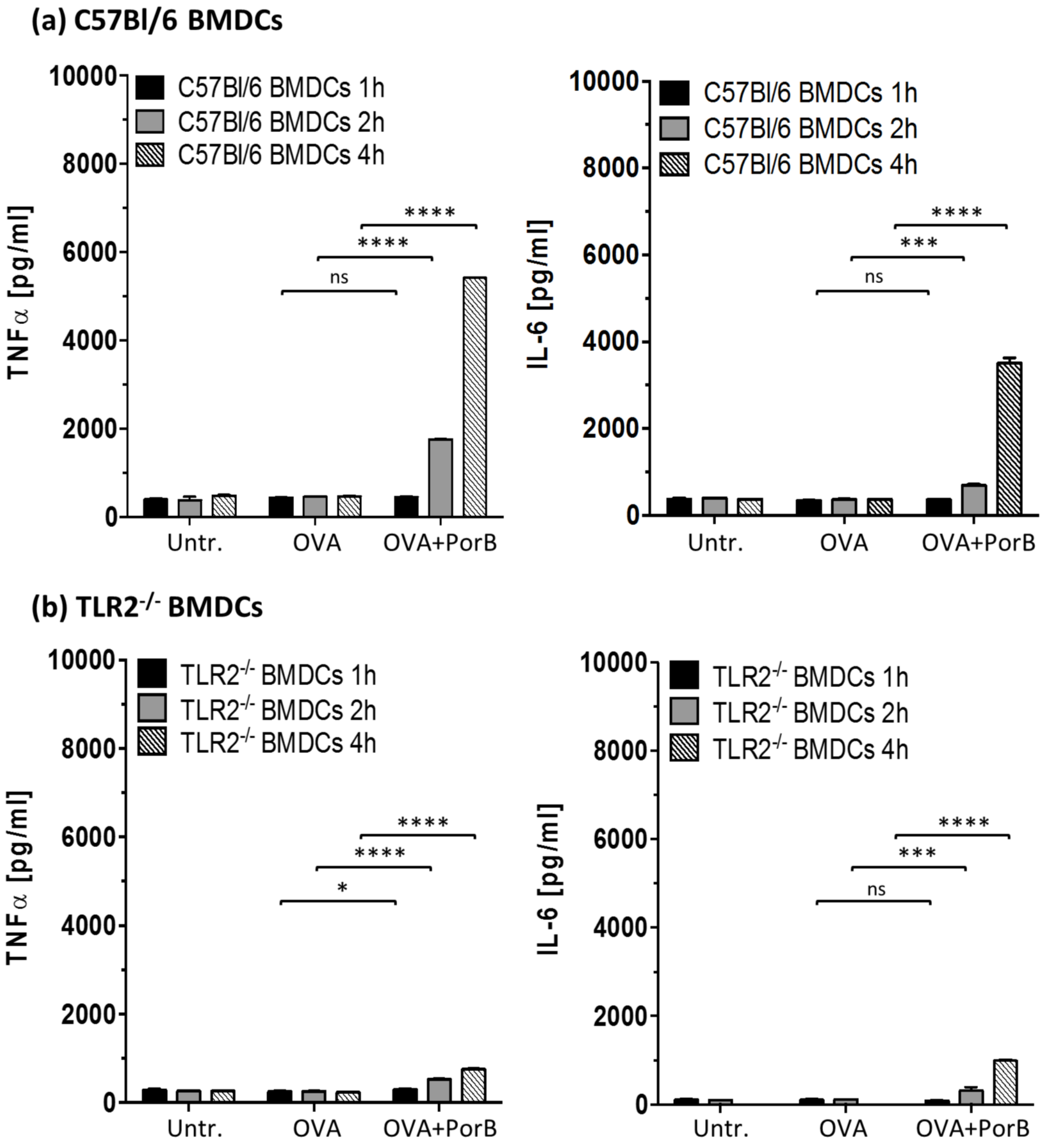

Supplemental Figure S3. Cytokine production by stimulated BMDCs isolated from either C57Bl/6 or TLR2$^{-/-}$ mice.

In vitro generated BMDCs derived from C57Bl/6 **(a)** or TLR2$^{-/-}$ mice **(b)** were left untreated (untr.) or were stimulated OVA protein or OVA+PorB for 1h, 2h or 4h and treated with Mitomycin C to prevent proliferation of BMDCs. Figure shows cytokine production in supernatant of BMDCs prior to coculture setup analyzed using TNFα and IL-6 ELISA. One out of 2 independent representative experiments is shown. Statistics were calculated using a Two-way

non-parametrical ANOVA with Tukey correction for multiple comparisons. ns $P > 0.05$, * $P < 0.05$, **$P < 0.01$, *** $P < 0.001$, ****$P < 0.0001$.

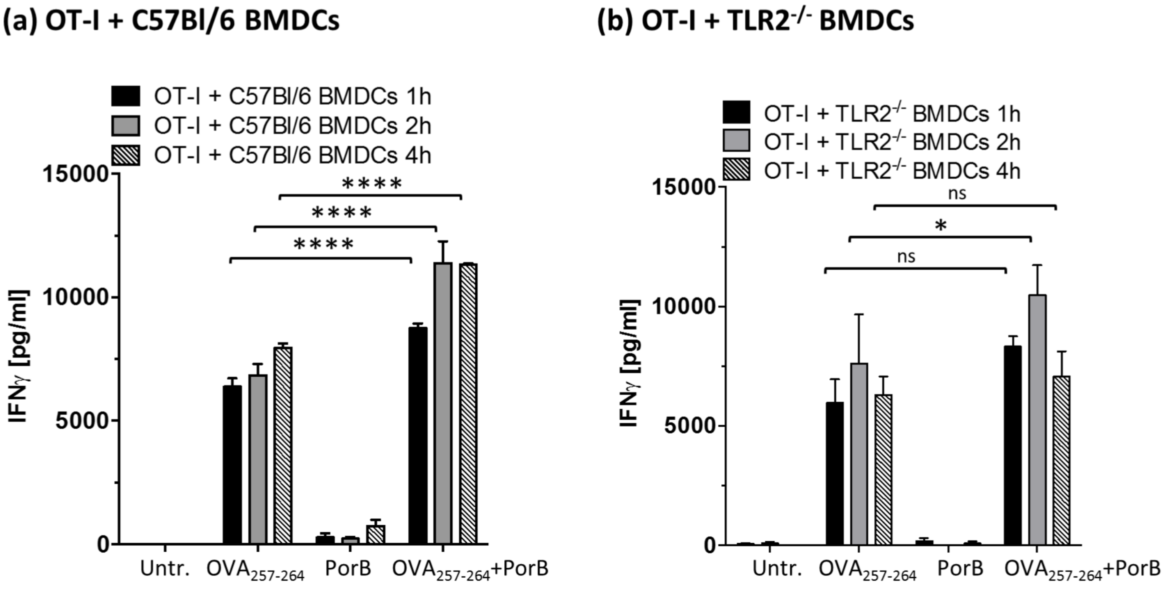

Supplemental Figure S4. Effect of PorB formulation on peptide stimulation of BMDCs cocultivated with OT-I splenocytes

In vitro generated BMDCs derived from wt **(a)** or $TLR2^{-/-}$ mice **(b)** were left untreated (untr.), or were stimulated with $OVA_{257\text{-}264}$ CD8 T cell peptide ("*SIINFEKL*"), PorB alone or $OVA_{257\text{-}264}$+PorB for 1h, 2h or 4h respectively and treated with Mitomycin C. After removal of superantant and extensive washing, BMDCs were cocultivated with OT-I splenocytes for 3 days in an effector to target ratio of 4:1. IFNγ production was determined as a readout for antigen recognition by OT-I splenocytes using ELISA. One out of 2 independent representative experiments is shown. Statistics were calculated using a Two-way non-parametrical ANOVA with Tukey correction for multiple comparisons. ns $P > 0.05$, * $P < 0.05$, **$P < 0.01$, *** $P < 0.001$, ****$P < 0.0001$.

(a) OT-II + C57Bl/6 BMDCs **(b) OT-II + TLR2$^{-/-}$ BMDCs**

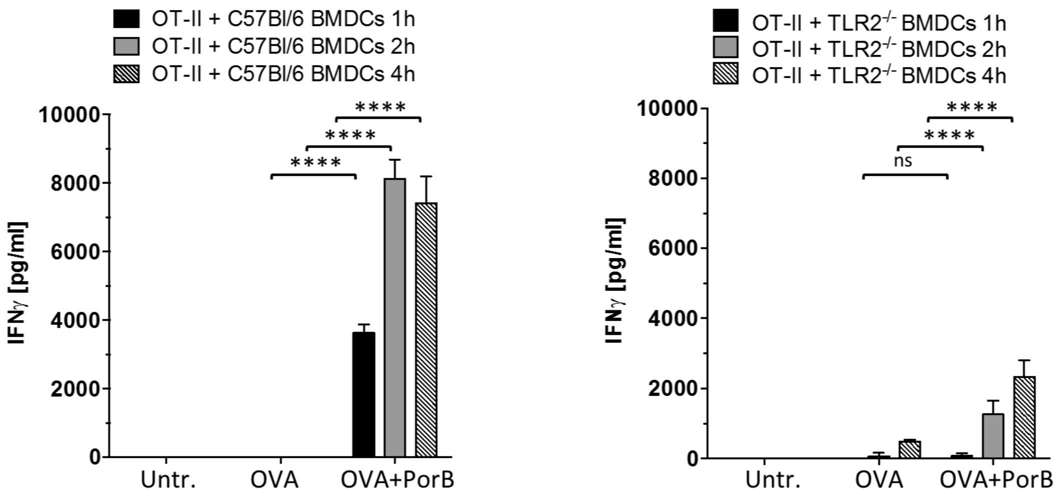

Supplemental Figure S5. PorB enhances antigen presentation of BMDCs to OT-II splenocytes. *In vitro* generated BMDCs derived from either C57Bl/6 **(a)** or TLR2$^{-/-}$ mice **(b)** were left untreated (untr), or were stimulated with OVA or OVA+PorB for 1h, 2h or 4h and treated with Mitomycin C to prevent proliferation of BMDCs. Figure shows IFNγ levels in supernatant of OT-II - BMDC cocultures. One out of 2 independent representative experiments is shown. Statistics were calculated using a Two-way non-parametrical ANOVA with Tukey correction for multiple comparisons. ns P > 0.05, * P < 0.05, **P < 0.01, *** P < 0.001, ****P < 0.0001.

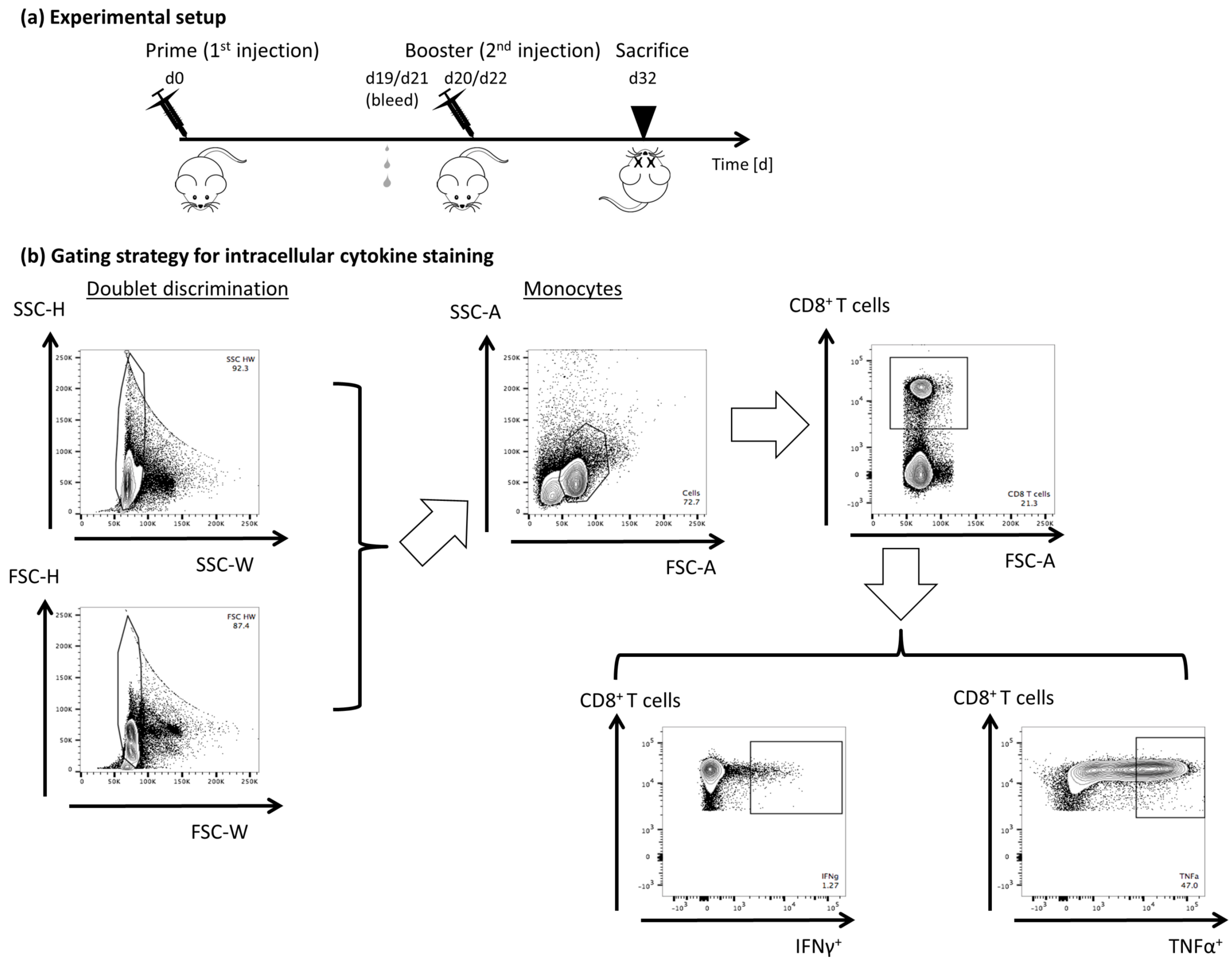

Supplemental Figure S6. Mouse vaccination setup and flow cytometry gating strategy for *ex vivo* stimulated splenocytes and intracellular cytokine staining.

(a) Mouse vaccination setup: mice received 2 injections with OVA or OVA+PorB 20 days apart. Mice were sacrificed 10 respectively 12 days post booster vaccination and single cell splenocytes suspension prepared and *ex vivo* restimulated with indicated peptides. **(b)** Gating strategy for *ex vivo* restimulated splenocytes. Splenocytes were stimulated *ex vivo* for 4h with CD3/CD28 antibodies [3 µg/ml] as positive control and is shown here.

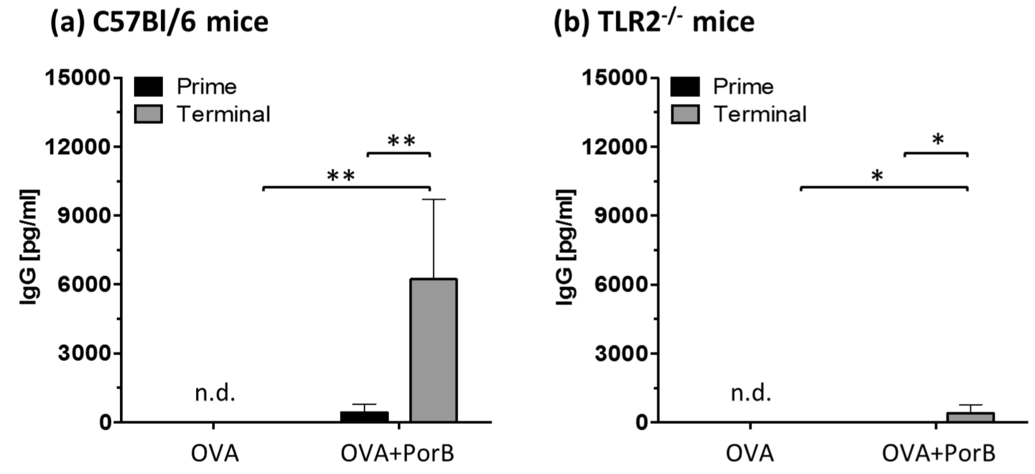

Supplemental Figure S7. Humoral immune response against OVA in C57Bl/6 and TLR2$^{-/-}$ mice triggered through PorB formulation.

Wt **(a)** and TLR2$^{-/-}$ **(b)** were immunized intramuscularly (i.m.) with either OVA or OVA+PorB and received a booster vaccination 20 days later into the *tibialis anterior* muscle. Humoral immune response was determined from serum obtained prior to booster vaccination (prime) and 12 days post booster vaccination (terminal) using OVA specific IgG ELISA. One representative experiment of wt and TLR2$^{-/-}$ vaccinated mice is shown out of 2 independent experiments. Statistics based on 2-way ANOVA with Tukey's multiple comparisons test. n.d. below threshold, ns P > 0.05, * P < 0.05, **P < 0.01.

PorB polyacrylamide gel electrophoresis

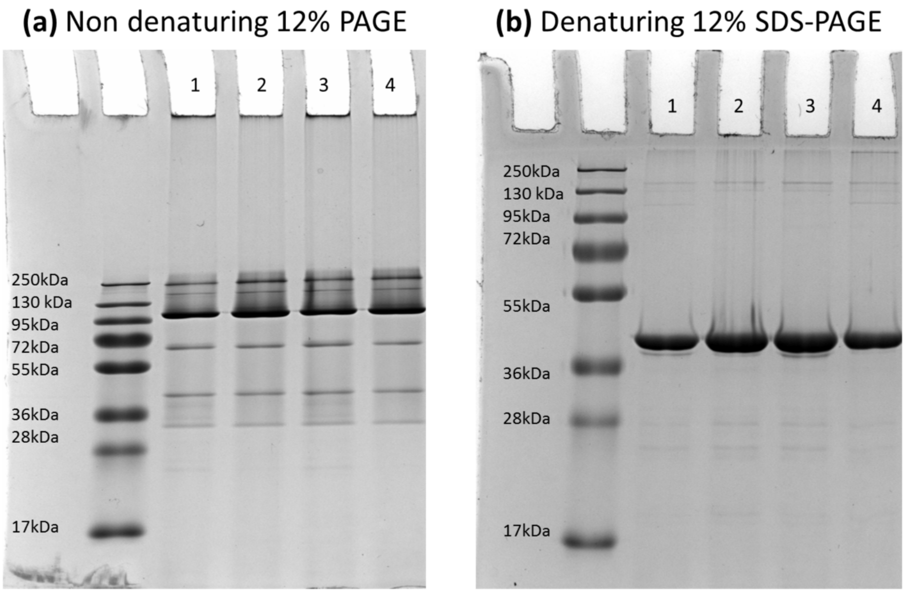

Supplemental Figure S8. Polyacrylamid gelelectrophoresis of purified *Neisseria meningitidis* PorB.

Samples 1-4 show different isolations of *N. meningitidis* PorB preparations 10 µg of protein per lane loaded per sample and 8 µl of marker loaded (Thermo Scientific, PageRuler Plus). Different PorB preparations were analyzed using non-denaturing (**a**) and denaturing (**b**) 12% polyacrylamide gelelectrophoresis (PAGE). Non-denaturing PAGE samples were separated on a 12% PAGE with sample loading buffer containing 20% glycerol and bromphenolblue. Denatured SDS-PAGE was conducted boiling samples for 5 minutes in β-mercaptoethanol (5%) and SDS containing loading buffer. Gels were stained in CoomassieBlue staining solution for 1h and afterwards destained using destaining solution (10% methanol, 10% isopropanol, 80% ddH_2O). Images were acquired on a Biorad Gel Doc XR imaging system (Biorad, USA).

	TNFα		IL-6		IFNβ	
Sidak's multiple comparisons test	Significant?	Summary	Significant?	Summary	Significant?	Summary
**0.5h**						
OVA-A594 vs. ctrl	No	ns	No	ns	No	ns
OVA-A594 vs. OVA-594+PorB	No	ns	No	ns	No	ns
OVA-A594 vs. OVA-594+Pam3CSK4	No	ns	No	ns	No	ns
OVA-A594 vs. OVA-594+LPS	No	ns	No	ns	No	ns
OVA-A594 vs. OVA-594+CpG	No	ns	No	ns	No	ns
**2h**						
OVA-A594 vs. ctrl	No	ns	No	ns	No	ns
OVA-A594 vs. OVA-594+PorB	No	ns	No	ns	No	ns
OVA-A594 vs. OVA-594+Pam3CSK4	Yes	****	No	ns	No	ns
OVA-A594 vs. OVA-594+LPS	Yes	****	No	ns	No	ns
OVA-A594 vs. OVA-594+CpG	No	ns	No	ns	No	ns
**4h**						
OVA-A594 vs. ctrl	No	ns	No	ns	No	ns
OVA-A594 vs. OVA-594+PorB	Yes	****	No	ns	No	ns
OVA-A594 vs. OVA-594+Pam3CSK4	Yes	****	No	ns	No	ns
OVA-A594 vs. OVA-594+LPS	Yes	****	No	ns	Yes	****
OVA-A594 vs. OVA-594+CpG	Yes	****	No	ns	No	ns
**6h**						
OVA-A594 vs. ctrl	No	ns	No	ns	No	ns
OVA-A594 vs. OVA-594+PorB	Yes	****	No	ns	No	ns
OVA-A594 vs. OVA-594+Pam3CSK4	Yes	****	Yes	**	No	ns
OVA-A594 vs. OVA-594+LPS	Yes	****	Yes	***	Yes	****
OVA-A594 vs. OVA-594+CpG	Yes	****	No	ns	Yes	****
**8h**						
OVA-A594 vs. ctrl	No	ns	No	ns	No	ns
OVA-A594 vs. OVA-594+PorB	Yes	****	Yes	****	No	ns
OVA-A594 vs. OVA-594+Pam3CSK4	Yes	****	Yes	****	No	ns
OVA-A594 vs. OVA-594+LPS	Yes	****	Yes	****	Yes	****
OVA-A594 vs. OVA-594+CpG	Yes	****	Yes	****	Yes	****

Table 1. Statistical analysis of TNFα, IL-6 and IFNβ cytokine production by BMDCs incubated with PBS, OVA or OVA+PorB.

Statistics based on 2 way ANOVA with Sidak’s correction for multiple comparisons, ns $P > 0.05$, * $P < 0.05$, **$P < 0.01$, *** $P < 0.001$, ****$P < 0.0001$.

Target	Host	Color	Clone	Supplier
CD11c		APC	HL3	BD
CD11c		PE	HL3	BD
CD11b		APC-Cy7	M1/70	Biolegend
CD11b		FITC	M1/70	BD
CD8		APC-Cy7	53-6.7	Biolegend
mouse-IFNγ		FITC	XMG1.2	Biolegend
mouse-TNFα		APC	clone cc MP6-XT22	Biolegend
EEA1	goat	-	C15	SantaCruz
LAMP1	rat	-	1D4B	Biolegend
rat-IgG	chicken	Alexa-488		Life Technologies
goat-IgG	donkey	Alexa-647		Life Technologies

Table 2. List of antibodies used for flow cytometry and immunofluorescence microscopy.

Supplementary Protocols

PorB Purification

PorB was purified from *N. meningitidis* strain H44/76 Δ-1/4 [1] using protein extraction and column chromatography as previously described [2]. PorB was formed into protein micelles, termed proteosomes, for vaccine preparation or *in vitro* cell stimulations, as previously described [3,4]. Purified PorB was tested to stimulate TLR1/TLR2 expressing HEK cells as described previously [5]. Endotoxin content was determined using LAL assay (Pierce Thermo Scientific, USA) as described [6]. PorB preparations (10 µg per lane) were analyzed using non-denaturing and denaturing 12% polyacrylamide gel electrophoresis (PAGE). Non-denaturing PAGE samples were separated on a 12% PAGE with sample loading buffer containing 20% glycerol and bromophenol blue. Denatured SDS-PAGE was conducted on samples boiled for 5 minutes in β-mercaptoethanol (5%) SDS containing loading buffer. Gels were stained in Coomassie Blue staining solution for 1h and afterwards de-stained using de-staining solution (10% methanol, 10% isopropanol, 80% ddH_2O). Images were acquired on a Biorad Gel Doc XR imaging system (Biorad, USA).

Cell stimulation and antigen uptake assays

BMDCs derived from C57Bl/6 mice were plated in 96U-well plates (Corning) at $5x10^5$ cells/ml. BMDCs were rested for 1h before Ovalbumin Alexa Fluor-594 Conjugate (referred to as OVA-A594) (Invitrogen, #O34783) was added to the wells at 5 µg/ml formulated with the indicated TLR ligand as adjuvant, i.e. 100 ng/ml *E. coli* strain O111:B4 LPS (LIST Biological Laboratories,

USA), 100 ng/ml Pam_3CSK_4 (Invivogen, #tlrl-pms), 10 µg/ml *N. meningitidis* PorB, 1 µg/ml CpG (Invivogen, #tlrl-1826-1) or serum free media (UltraCulture Lonza, # 12-725F) only. Cells were incubated for 0.5h, 2h, 4h, 6h or 8h respectively. After stimulation supernatant was collected, stored at -20°C for Cytokine ELISA and BMDCs were stained for flow cytometry as described below. For confocal images, $5x10^5$ cells were seeded on day 6 on fibronectin (Neuvitro, Germany, #GG-12-fibronectin) coated cover slips and rested over night before stimulation with fluorescently labeled OVA-A594 [5 µg/ml] with or without PorB formulation [10 µg/ml].

Generation of single cell suspension of spleens and lymph nodes

Spleens and/or lymph nodes from either OVA transgenic mice (OT-I/OT-II) or vaccinated mice (C57Bl/6 or $TLR2^{-/-}$) were harvested and a single cell suspension was prepared as follows. Briefly, spleens were pushed through a 70 µm cell strainer. Red blood cells in samples derived from spleens were lysed using ACK lysis buffer for 3 minutes, washed in PBS and afterwards *ex vivo* stimulated with peptides or cocultured with BMDCs, in the case of OT-I/OT-II splenocytes. Lymph nodes were torn with tweezers and digested using 30 µl of 1 mg/ml Collagenase D (ThermoFisher, USA) in 1 ml RPMI-1640 for 30 minutes at 37°C with gentle agitation to allow easier separation of dendritic cells. Single cell suspension was generated by grinding the remaining tissue through a 70 µm nylon mesh. Cells were washed in PBS, counted and stained for analysis using flow cytometry. Briefly, cells were incubated for 15 minutes with CD16/CD32 F_c block and afterwards stained for 30 minutes with CD11b – APC-Cy7 (Biolegend, USA), CD11c – APC (BD, USA). Antibodies were diluted 1:200 unless otherwise indicated.

Immunoglobulin ELISA

OVA specific immunoglobulin (IgG) levels were determined using Enzyme-linked Immunosorbent Assay (ELISA). Briefly, Immunolon 2HB Microtiter ELISA plates were coated with OVA (5 µg/ml) in carbonate buffer and incubated overnight at 4°C. Sera were diluted starting at 1:50 and incubated overnight at 4°C. Alkaline phosphatase-conjugated anti-mouse IgG (Sigma-Aldrich, USA) was used to detect IgG. ELISA was developed with one-step p-nitrophenyl phosphate (Pierce, USA) and the optical density (OD) at 405 nm was measured on a SpectraMax190 Microplate Reader (Molecular Devices, Sunnyvale, CA). IgG level were determined using standard curves generated by known amounts of IgG using GraphPad Prism (version 6.0).

Additional References Supplementary Figures and Supplementary Protocols

1 Mackinnon, F. G. *et al.* The role of B/T costimulatory signals in the immunopotentiating activity of neisserial porin. *The Journal of infectious diseases* **180**, 755-761, doi:10.1086/314966 (1999).

2 Langley, J. M. *et al.* Safety and immunogenicity of a Proteosome -trivalent inactivated influenza vaccine, given nasally to healthy adults. *Vaccine* **24**, 1601-1608, doi:10.1016/j.vaccine.2005.09.056 (2006).

3 Wetzler, L. M., Blake, M. S., Barry, K. & Gotschlich, E. C. Gonococcal porin vaccine evaluation: comparison of Por proteosomes, liposomes, and blebs isolated from rmp deletion mutants. *The Journal of infectious diseases* **166**, 551-555 (1992).

4 Reichardt, P., Dornbach, B. & Gunzer, M. APC, T cells, and the immune synapse. *Current topics in microbiology and immunology* **340**, 229-249, doi:10.1007/978-3-642-03858-7_12 (2010).

5 Toussi, D. N. *et al.* The amino acid sequence of Neisseria lactamica PorB surface-exposed loops influences Toll-like receptor 2-dependent cell activation. *Infect Immun* **80**, 3417-3428, doi:10.1128/IAI.00683-12 (2012).

6 Liu, X., Wetzler, L. M. & Massari, P. The PorB porin from commensal Neisseria lactamica induces Th1 and Th2 immune responses to ovalbumin in mice and is a potential immune adjuvant. *Vaccine* **26**, 786-796, doi:10.1016/j.vaccine.2007.11.080 (2008).
